# Developing Indicators to Measure Critical Health Literacy in the Context of Norwegian Lower Secondary Schools

**DOI:** 10.3390/ijerph19053116

**Published:** 2022-03-07

**Authors:** Anders L. Hage Haugen, Kirsti Riiser, Marc Esser-Noethlichs, Ove Edvard Hatlevik

**Affiliations:** 1 Faculty of International Studies and Teacher Education, Oslo Metropolitan University, 0130 Oslo, Norway; marno@oslomet.no (M.E.-N.); ove-edvard.hatlevik@oslomet.no (O.E.H.); 2 Faculty of Health Science, Oslo Metropolitan University, 0130 Oslo, Norway; kiri@oslomet.no

**Keywords:** critical health literacy, health literacy, adolescents, school, health education, lower secondary schools, health and life skills, well-being

## Abstract

A critical health literacy (CHL) approach is recommended for promoting health in the school context. This construct is complex and includes three interconnected domains: (A) appraisal of critical information, (B) awareness of the social determinants of health (SDH), and (C) collective action to promote health and well-being. In recent literature reviews, no measurement instrument that covers all three domains of CHL in the school-context was found. Our aim was to develop self-reported measurement scales for each domain of CHL. The development process reported in this study was conducted in two stages. In the first stage, an initial item pool was generated based on literature reviews and focus group interviews (N = 15) with adolescents (steps 1–2). In the next steps, items were adjusted and removed based on the feedback from an expert panel and from representatives from the target group (steps 3–5). In stage two, we aimed to reduce the number of items and develop scales for each domain. We then piloted the current draft, which consists of 28 items (N = 114). A sub-sample (N = 10) of the participants were interviewed after they completed the survey to examine the instrument’s face validity. Cronbach’s α was used to assess the internal reliability of the scales; the reliability was promising for scales A (α = 0.83) and C (α = 0.85) but was below the recommended value for scale B (α = 0.61). The model fit indices were promising (TLI_scaleA_ = 0.97, RSMEA_scaleA_ = 0.055, TLI_scaleB_ = 1.05, RMSEA_scaleB_ = 0.00, TLI_scaleC_ = 0.95, RMSEA_scaleC_ = 0.074). The piloted version of scales A and C were positively correlated with subjective health literacy, health-related quality of life, and subjective health; however, we found no such correlations for scale B. The post-survey group interviews led to some adjustments in scales A and B. The revised version of CHLA-Q must be tested using a larger sample; this will enable more robust statistical testing of the properties of the items and the scale.

## 1. Introduction

Health Literacy (HL) has become a crucial goal of health education efforts across the globe [1,2,3,4]. Adolescents frequently engage with health-related information provided by their peers, adults, the media, social media, and commercial forces [1]. During the recent COVID-19 pandemic, the processing of health information has become even more important for individual and collective health. Considering the recent pandemic, researchers have emphasized the need to see HL in relation to social responsibility and solidarity [5]. Schools are central actors in this area and can help to improve HL and connect it to social responsibility among all adolescents across diverse social groups [1,4,6,7]. According to the Norwegian Education Act [8], children and adolescents should gain knowledge, skills, and attitudes that will help them to master their lives and participate in and contribute to adult life and the communities in society. A recent update to the national curriculum introduces a new cross-curricular subject, Health and Life Skills (HLS) [9]. This subject focuses on such skills and connects them to health and well-being. HLS has been introduced due to concerns about the health of children and adolescents; this part of the curriculum aims at helping school-aged children to develop competencies that contribute to obtaining and sustaining health and well-being throughout their adolescence and the rest of their lives. In Norway, the term ‘health literacy’ is not explicitly used in the curriculum; however, the subject of HLS aligns well with contemporary frameworks and recommendations related to HL. A recent review of school-based HL interventions emphasize that HL should be taught across different subjects and using an inquiry-based and critical learning strategy [4]; these approaches correspond with the purpose and content in the cross-curricular subject HLS.

Globally there is no consensus on a definition for adolescent or childhood HL [10]. However, HL is broadly accepted as a complicated, multifaceted, and multidimensional social construct [11,12]. It revolves around people’s abilities to find, understand, appraise, and apply health-related information to promote and sustain health and well-being throughout their lives [13,14,15,16]. HL depends on contextual conditions that restrict or enhance an individual’s opportunities [10,12,16,17], such as environmental demands and available resources. This highlights the need for the entire school and community to be involved in developing HL. At the individual level, it has been argued that HL efforts in schools should focus on teaching meta-cognitive skills, such as critical thinking, self-awareness, and citizenship, rather than on the transfer of theoretical and practical knowledge [18]. In addition, HL education should draw attention to the social determinants of health and the social power relations responsible for health inequities [18]. Expanding pupils’ understanding of health beyond individual actions to social and structural factors can help to reduce the individualistic focus that some HL approaches have been criticized for [6,16]; a concern that has also been raised regarding the introduction of HLS [19]. Thus, a critical approach to HL is frequently recommended for school contexts [6,20].

Critical health literacy (CHL) as an aspect of HL, was inspired by literacy research where critical literacy is defined as “more advanced cognitive skills which, together with social skills, can be applied to critically analyze information, and to use this information to exert greater control over life events and situations” [11]. Chinn [21] elaborates on this and suggests that CHL is a unique concept that includes the following three interconnected domains: (1) information appraisal, (2) understanding the social determinants of health, and (3) competencies that enable actions for the promotion of collective health [21,22,23]. Little research has been performed on CHL in a school context. In a relatively recent literature review, only six school-based CHL studies were identified [24]. This review further revealed that evaluative findings are rarely reported and found no instrument for measuring CHL as a distinct construct in schools [24]. However, CHL is often operationalized as one level of a multidimensional HL construct. Even though the HL of adolescents has received less attention than that of adults, there is a growing body of research in this area.

### 1.1. Measures of HL for Adolescents

As of this writing, four comprehensive literature reviews of HL instruments for adolescents have been published [25,26,27,28]. Among the measures included in these reviews are five highly relevant generic instruments that address aspects of CHL in adolescents (aged 10–17 years), which are available in English [29,30,31,32,33]. Two additional instruments for adolescents have been published after the most recent review [34,35]. In the following section, a brief description of the seven instruments is given.

The health literacy measure for adolescents (HELMA) was developed for adolescents aged 15–18 years old in Iran [30]. It is a multidimensional comprehensive measure with eight factors and 44 items, including five self-reported items that measure the information appraisal domain. Another, much shorter instrument, is the HLAT-8 [29]. This was developed in Switzerland and specifically targets HL for family and private contexts. The measure includes eight items across three theoretical dimensions, four items measuring functional HL, two items for interactive HL, and two items for critical HL in terms of information appraisal. The health literacy assessment scale for adolescents (HAS-A) measures HL in the following three factors: communicating health information, confusion about health information, and understanding health information [31]. The measure focuses on the health care setting, but also includes items more related to health promotion.

Two instruments were made specifically for the school context. One of which is an exclusively performance-based instrument for measuring HL in Canadian high school students [33]. They identified some criteria that adolescents can use to judge the credibility of health information, among them are the following: (1) accuracy, (2) impartiality, and (3) relevance. Another measure that was created for the school context is the health literacy for school-aged children (HLSAC) [32]. It is based on the theoretical framework of HL as learning outcomes for the Finish school context [1]. HLSAC measures subjective HL with ten items in one factor with five theoretically distinct components. These are ordered from the least complex to more advanced as follows: (1) theoretical knowledge, (2) practical knowledge, (3) individual critical thinking, (4) self-awareness, and (5) citizenship. Theoretically, the components of self-awareness and citizenship are related to the third domain of CHL, namely collective action for health. Within this framework, citizenship evolves around the abilities to understand and act upon both the rights and responsibilities that comes with participation in a democratic collective.

Recently, two more instruments have been developed and adapted from the European Health Literacy Survey Questionnaire framework (HLS-EU-Q) [15]. The Measurement of Health Literacy Among Adolescents Questionnaire (MOHLAA-Q) is an age-adjusted generic instrument developed for adolescents aged 14–17 years in Germany [35]. The final version consisted of four scales with 29 items in the following three areas: (A) dealing with health-related information, (B) interaction and communication skills, and (C) attitudes towards one’s own health and health information. Critical appraisal is measured through three items in scale A. HLS-Child-Q15 was adapted for children aged 9–10 years [34]. A selection of items from HLS-EU-Q was modified and distributed to a sample of German children aged 9–10 years. The final instrument consisted of 15 items measuring generic HL, of which one item was intended to measure the appraisal dimension relatable to the first domain of CHL.

For the most part, these studies address the first domain of CHL, namely critical information appraisal. However, the other two domains—understanding the social determinants of health and collective action for health and well-being—are not explicitly addressed in existing HL measures for adolescents.

### 1.2. Aims and Structure

Along with Sykes and Wills [24], we claim that there is no suitable measurement tool that accounts for all three domains of CHL for use among adolescents in lower secondary schools. The purpose of the present study was to develop scales to measure all three domains of CHL. The specific aims were (1) to explore and develop self-reported indicators of CHL, (2) to test these indicators in a sample of adolescents in lower secondary schools in Norway before (3) revising the indicators based on item analysis, exploratory factor analysis, and post-survey focus group interviews. Following the recommendations for HL research [7,26], we used both qualitative and quantitative approaches and involved the target group in the development process. We drew on previous research and adapted items from existing instruments; we also created new items throughout the development phase.

The development process is complex and includes several interconnected steps. To provide a straightforward reading of the paper, these steps are described chronologically in the methods and results section.

## 2. Materials and Methods

### 2.1. Design

**Figure 1:**
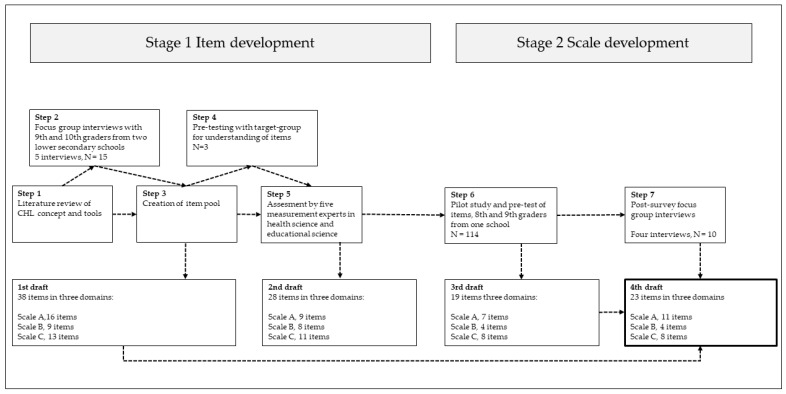
Stages of the study. Scale A: Critical Information Appraisal; Scale B: Awareness of Social Determinants of Health; Scale C: Citizenship for Health and Well-Being in the School Context.

This study reports on the development of three scales in a questionnaire measuring adolescents’ CHL, named the Critical Health Literacy for Adolescents Questionnaire, CHLA-Q (Figure 1). In health research, such scales can be developed in the following three phases: item development, scale development, and scale evaluation [36]. The present paper reports the first two stages. Stage 1, item development, consisted of a literature review (step 1), focus group interviews (step 2), and pre-tests with experts and representatives of the target group (steps 4–5). In Stage 2, we piloted the second draft of the CHLA-Q and investigated the psychometric properties of items and of scales A–C (step 6). Next, we conducted four post-survey group interviews to further examine the scale’s face validity (step 7). The results from step 7 were used to revise the scales.

### 2.2. Stage 1: Item Development

#### 2.2.1. Literature Review

The LR is summarized in the introduction of this paper because it is part of the rationale behind the aims of the study. The articles identified in the LR were read in detail and used for inspiration to adapt and write new items throughout the development process. No further analysis of the articles was performed for the purpose of this study. More details on the LR and included studies are given in the Supplementary Materials Table S2. References [1,2,3,4,5,6,7] are cited in the Supplementary Materials.

#### 2.2.2. Focus Group Interviews (Step 2)

Interviews were conducted during the spring of 2020 in two lower secondary schools in Norway. The teachers of one class from each school were recruited through school management, and the teachers distributed written consent forms to their students and parents. A total of ten ninth-grade students and six tenth-grade students agreed to participate (with their parents’ consent) (N = 15, N_boys_ = 4, N_girls_ = 11). Five group interviews with groups of two to four pupils were conducted. These interviews lasted 45 to 65 minutes. The aims of the interviews were (1) to explore adolescents’ experiences with and knowledge of the domains of CHL, (2) to assess the comprehensibility and perceived relevance of the questionnaire items (derived from the literature review of previous measures), and (3) to revise the items and write new ones based on these data. A theory-based approach was used in the semi-structured interviews. During the interviews, participants and interviewers worked together to create mind maps to explore participants’ current understanding of the social determinants of health. These conversations were recorded and transcribed before the analysis.

HyperRESEARCH™ (Version 4.2.5, Research Ware Inc., Randolph, MA, USA. 1988–2018) was used to structure the transcribed data for the qualitative analysis. A template approach [37] was used; a priori categories based on the theoretical framework of CHL formed the structure for the analysis. Meaningful and relevant passages in the interview were coded in appropriate domains and sub-categories and were used to inform item development. In this phase, we generated a first draft of the CHLA-Q consisting of 38 items.

#### 2.2.3. Pre-Testing (Steps 4–5)

To enhance content validity, these items were then examined by experts and by representatives from the target population [36,38]. The first draft of the items was sent in paper form to three adolescents. They were asked to mark or comment on any question they did not understand or found difficult or irrelevant. Three items were adjusted based on their feedback. An expert panel (N = 5) responded to a digital survey that enabled comments on the scales and items. The expert panel consisted of three researchers in the field of health science and two researchers in the field of educational science. A definition was provided for each domain. The experts rated the items using a three-point Likert scale (3: essential; 2: useful but not essential; 1: unnecessary). All comments and feedback were carefully considered, and decisions to adjust or modify items were made by the primary author, as the scale developer [38]. A total of ten items were removed because they were rated unnecessary by at least two experts, and several others were adjusted based on the experts’ comments. The second draft consisted of 28 items in three domains.

### 2.3. Stage 2: Scale Development

#### 2.3.1. Cross-Sectional Survey (Step 6)

The second draft of the CHLA-Q was distributed to one school for pilot testing. This school is a participant in the Literacies for Health and Life Skills project [39], which includes the present study. A written consent form was distributed to eighth- and ninth-grade pupils and their parents. The digital survey took between 15 and 30 min to complete and was conducted during school hours. The researcher (primary author) was present, and all classes were given a standardized introduction about the survey. To collect information on pupils’ understanding of the different items, the participants were given the opportunity to ask questions and engage in short conversations with the researcher when needed.

#### 2.3.2. Analysis of Psychometric Properties (Step 6)

The aim of this analysis was to examine the psychometric properties of the items and the scales and to reduce the total number of items without compromising the internal reliability or validity of the scales. Each item was analyzed with respect to item difficulty, variance, and corrected item–scale correlation (ITC). ITC was computed by calculating Pearson’s correlation among item scores and the remaining sum score within each scale [38]. Item difficulty was assessed by reviewing the means, skewness, and response distribution for each item. To ensure sufficient variance, we also investigated to what extent all categories were used.

Exploratory factor analysis (EFA) was conducted on each domain separately to ensure at least ten respondents per item [40,41]. The Kaiser–Meyer–Olkin measure of sampling adequacy (MSA) was used to determine whether the data was suited for factor analysis within each scale, and an overall MSA greater than 0.50 was accepted [42,43]. Principal axis factor analysis, with maximum likelihood factor calculation was performed, and parallel analysis was applied to determine number of factors to extract [41]. Oblique rotation, which allows for correlations among factors, was used to facilitate interpretation and to determine the best factor structure [40]. Items with factor loadings below 0.30 and items that cross-loaded were removed one at a time. When items were removed, internal reliability and improvement in fitness indices were considered. The Tucker–Lewis index of factor reliability (TLI > 0.95) and the root mean square of residuals (RSMEA < 0.08) were used as indicators of how well the factor model fitted the empirical data [44]. A Cronbach’s α of 0.70–0.95 was the desired reliability for each factor [45]. However, when measuring complex constructs, such as CHL, internal reliability may be obtained at the expense of content validity, as some degree of heterogeneity in items is necessary to fully examine the phenomenon of interest [45]. When interpreting and labelling the factors, we emphasized that the items associated with each factor should make sense according to the theoretical background and qualitative data retained in the earlier steps of the study [38].

To test convergent validity, we hypothesized that scales A–C should moderately correlate with general subjective HL, health-related quality of life (HrQoL), and subjective health. Confirmation of pre-determined assumptions is an indication of convergent validity [45]. HL was measured using the HLSAC [32], a ten-item subjective measure of generic HL that was developed in Finland and has been tested in several European countries [46]. HLSAC has been translated to Norwegian [47] and used in previous studies of Norwegian adolescents (aged 16–19 years) [47,48]. Scores were summed for a total HLSAC score ranging from 10 to 40. HrQoL was measured using the Norwegian version of Kidscreen-10, a shorter version of the more comprehensive Kidscreen-52 instrument [49]. Subjective health was measured with a single question about the participant’s health status (1: bad; 2: fairly good; 3: good; 4: very good; 5: excellent). Subjective health has been established as an important health indicator and a predictor of future health outcomes [50,51].

All statistical analysis were performed using RStudio; the ‘psych’ package [52] was used for EFA and to investigate the internal reliability and convergent validity (correlation tests).

#### 2.3.3. Post-Survey Focus Group Interviews (Step 7)

Interviews were conducted immediately after the participants had completed the survey. These interviews were used to evaluate how well members of the target group understood the items and categories and whether they measured the intended domains of the study [36]. Three interviews (N = 10), each lasting approximately 30 min, were conducted with the eighth- and ninth-grade participants. The survey items were used to guide the semi-structured interviews. The interviewees were first asked to talk about words or questions that they thought were difficult or strange. Then the researcher asked more specific questions about each scale, including questions about the participants’ interpretations of response categories. All scales were discussed, but not all items. The perspectives of the target group were emphasized during the development of the fourth draft of the CHLA-Q (step 7). The results of the psychometric analysis were considered together with the feedback from the target group. This enabled informed adjustments to items that performed poorly in the psychometric analysis.

### 2.4. Ethical Considerations

All steps of this study were approved by the Norwegian Centre for Research Data (NDS, ref: 313927 & 186349) before data collection was initiated. Services for sensitive data (TSD) were used to store digital data and ensure safe processing. Minimal identifiable data were collected, and broad categories of sociodemographic variables were used to ensure anonymity. Teachers distributed and collected written consent forms from all participants and their parents. On the day of the survey, it was emphasized that participation was voluntary.

## 3. Results

The results are presented in three sections. The first section includes the results of the item development (stage 1) and outlines the process that resulted in the second draft of the CHLA-Q. The second section reports the results of the quantitative testing of the second draft and the reduction in items that led to the third draft. The third section describes the re-examination and revising of scales A–C based on the post-survey group interviews and presents the fourth draft of the CHLA-Q.

### 3.1. Stage 1: Item Development

#### Focus Group Interviews (Step 1)

The focus group participants considered learning about health-related information-appraisal strategies to be meaningful and necessary. They reported knowing about several critical appraisal strategies, such as investigating the characteristics of the information (e.g., who wrote it, when it was written, what sources the author used, and how professional it looks) and comparing information with other sources. However, they said that they did not always use these strategies in everyday situations. Most participants agreed that searching the web for health information could lead to unwarranted anxiety. Thus, being able to judge the relevance of the information was an important theme for scale A. Moreover, the adolescents expressed concerns about their abilities to appraise health-related information; strong dependence on parental help to evaluate the information was consistent across all the groups. Based on these qualitative data and inspired by the existing measurements of generic HL in adolescents [30,31,32,33,34,35,36], items were generated for the first draft of scale A. Items addressing the ease or difficulty of asking for help were included, along with items on comparing sources, assessing the relevance of information to one’s own situation, and appraising the credibility of information. All the items began with “How easy or difficult is it for you to…”, and the response options were the following: very difficult (1), difficult (2), sometimes difficult (3), easy (4), and very easy (5).

To develop scale B, focus group participants and the interviewer created mind maps together and discussed the factors that the adolescents considered important for health and well-being. Most participants emphasized the significance of lifestyle factors (e.g., nutrition, physical activity, sleep). Social support was deemed highly important to mental health (e.g., having friends, being noticed/talked to). However, participants also expressed a deeper awareness of the social determinants of health. Topics, such as democratic participation and socio-economic factors, were mentioned. The participants also revealed some knowledge of health inequalities (e.g., cheap food often is less healthy). They were aware of health inequalities from a global perspective but had less insight on national or local inequalities. When asked about the relative importance of structural factors (e.g., housing, access to education) and lifestyle choices (e.g., nutrition, physical activity), most groups indicated that structural factors were more important. However, these responses might have been impacted by the context. These discussions were used to further develop items for scale B, addressing the relationship between individual and collective responsibility for health and health inequalities due to unequal opportunities. All of the items were introduced with “To what extent do you agree with the following statements?” The response options were as follows: completely disagree (1), disagree (2), agree (3) and completely agree (4).

The third domain (scale C) revolves around competencies that enables actions that promote health in a collective. Chinn [21] suggests that cooperation, empathy, and self-confidence might impact actions that promote health in a local environment; thus, these aspects were explored further. The adolescents mostly expressed concerns about social and mental well-being in the school context, and they reflected on how their own and their peer’s behavior (e.g., mood, smiles, comments) could affect their social environment. The participants had many suggestions for improving the structural supports that promote health and well-being (e.g., more control and predictability, fewer tests, more frequent breaks, and more physical activity). However, pupils’ experience with participating in decisions concerning these matters varied. Aligning with literature suggesting that citizenship is an integral part of health literacy [1], scale items were adapted to measure self-efficacy in social tasks related to acting democratically and with a sense of social responsibility to benefit health and well-being in a collective. All 11 items in scale C began with the following phrase: “To what extent are these statements correct for you? I am a person who…”. The response options were as follows: not at all (1), a little (2), quite well (3), very well (4), and to a large extent (5).

### 3.2. Stage 2: Scale Development (Step 6)

A total of 114 out of 212 (54%) eighth- (60%) and ninth-graders (40%) participated in step 6 of the study. The proportion of girls and boys were 62% and 38%, respectively, and 32% of participants reported a migration background, defined as pupils with at least one parent who was not born in Norway. All the participants stated that one or both of their parents were employed, and 65% reported that both parents had more than three years of higher education (22% did not know). Most of the participants (92%) reported that they planned to pursue at least three years of higher education.

A detailed table of the descriptive statistics for each item is provided in the Supplementary Materials Table S1. The mean values varied depending on the scale range; while scales A and C ranged from one to five, scale B had only four options. For scale A, the mean ranged from 2.96 to 3.68; for scale B, it ranged from 2.68 to 3.22; and for scale C, it ranged from 2.88 to 4.28. Most of the items had a slight negative skew; for two items, skewness exceeded −1 (chl20, chl26). For scales A and C, variance ranged from 0.52 to 1.09 (chl3, chl28), and for scale B, variance ranged from 0.28 to 0.68. For most items, all the categories were used; the exceptions are items chl11, chl12, and chl17 in scale B. For these three items, the lowest category (totally disagree) was not used at all. The ITC was positive and significant (*p* < 0.05) for most items; the exceptions were item chl10 (r = 0.06) and chl15 (r = 0.09). Generally, items in scale B had a poorer ITC than those in the other scales; the ITC for items in scale B ranged from 0.06 to 0.51. For scales A and C, the ITC ranged from 0.43 to 0.70.

#### 3.2.1. Scale A: Critical Information Appraisal

The Kaiser–Meyer–Olkin test yielded an overall MSA of 0.83, which indicates that the data were meritoriously suitable for factor analysis [42]. The factor analyses were conducted in three stages to reduce the number of items with poor loadings (<0.30) and of items that cross-loaded. The initial parallel analysis yielded two factors for the nine-item scale. Six items loaded on F1 (perceived ease of judging accuracy), and three items loaded on F2 (perceived ease of judging relevance and seeking help). The fit indices were below the recommended minimum for adequate model fit (TLI = 0.84, RMSEA = 0.12) [44]. In the next steps, we removed items chl3 and chl4 to achieve a better balance between factors and improve the fit indices. In the final EFA (Table 1), we extracted two factors with eigenvalues of F1 = 2.0 and F2 = 1.7 across seven items. All the variables loaded above 0.34 on one of the two factors that together explained 52% of the variation. The seven-item scale had a Cronbach’s α of 0.83 (α_F1_ = 0.80, α_F2_ = 0.75). The model fit indices improved throughout the three stages, and the final model had promising fit with a TLI above 0.95 and an RMSEA of 0.055 [CI = 90%, 0–0.132]. Two items (chl6 and chl1) showed substantial cross-loading, suggesting that adjustment might be needed. Theoretically, F1 can be described as perceived ease of judging whether the information is relevant and of seeking help and F2 as perceived ease of judging whether the information is accurate.

#### 3.2.2. Scale B: Awareness of Social Determinants of Health

The Kaiser–Meyer–Olkin test yielded an overall MSA of 0.62, indicating that the data were within a mediocre but acceptable range for factor analysis [42]. In the initial parallel analysis, we removed two factors; however, three of the items did not load sufficiently to any factor (<0.30), and the fit indices were below the recommended values. In the four successive steps, we removed items chl10, chl15, chl11, and chl17. At each step, factor loading, internal reliability, and fit indices were considered. The final EFA (Table 2) yielded one factor with an eigenvalue of 1.3. This factor explained 32% of the total variance, and item loadings ranged from 0.37 to 0.80. The fit indices were good (TLI = 1.05, RMSEA = 0.00 [0–0.16]. However, the internal consistency of the scale was below the recommended value [46] with a Cronbach’s α of 0.61. The one remaining factor measures awareness of social inequality in health.

#### 3.2.3. Scale C: Citizenship for Health and Well-Being in the School Context

The Kaiser–Meyer–Olkin test yielded an overall MSA of 0.86, indicating that the data were meritoriously suitable for factor analysis [42]. The initial parallel analysis suggested that two factors should be extracted. All the variables loaded above 0.40 to one factor; however, four of the items (chl21, chl27, chl19, and chl25) cross-loaded, which makes the interpretation of the factor structure difficult. Stepwise EFAs were conducted without these items in turn. However, chl25 was not excluded to ensure balance between the two factors. Thus, the final model (Table 3) included eight items and two factors with eigenvalues of 2.5 and 1.8. Together, these factors explained 51% of the variance between the variables. The fit indices for the factor model were promising (TLI = 0.95, RSMEA = 0.074 [0–0.13]), and the scale’s internal reliability was good (Cronbach’s α = 0.85; α_F1_ = 0.86, α_F2_ = 0.73). Theoretically, F1 can be described as self-efficacy in providing social support and F2 as self-efficacy in democratic participation to promote health and well-being.

#### 3.2.4. Convergent Validity

We found strong positive correlations between generic HL and scales A (r = 0.629, *p* < 0.001) and C (r = 0.715, *p* < 0.001). We found moderate positive correlations between HrQoL and scale A (r = 0.366, *p* = 0.007) and scale C (r = 0.308, *p* = 0.002). Furthermore, these scales correlated positively with the self-reported health status (r_scaleA_ = 0.220, *p* = 0.02; r_scaleC_ = 0.245, *p* = 0.01). However, when we examined correlations for each factor in scales A and C separately, we found that only the first factor (F1) in scale A correlated significantly with the subjective health status (r = 0.267, *p* = 0.004). The second factor (F2) did not correlate significantly with the subjective health status (r = 0.126, *p* > 0.05). For scale C, correlations with the subjective health status remained significant for both factors. However, the correlation of scale C’s second factor (F2) with HrQoL (r = 0.189, *p* > 0.05) was not significant. We did not find any significant correlations between scale B and HL, HrQoL, or the self-reported health status. Scales A and C were positively correlated with one another (r = 0.421, *p* < 0.001), while scale B did not correlate with scale A (r = −0.014, *p* > 0.05) or scale C (r = 0.021, *p* > 0.05).

### 3.3. Stage 2: Post-Survey Focus Group Interviews and Revision of the Third Draft (Step 7)

We used the results of the post-survey group interviews to make additional adjustments to the instrument. Respondents reported that the ease of judging the credibility of information depends on the type of information at hand. Therefore, we concluded that distinguishing health-promoting information from disease- or risk-reducing health information for each item would make it easier for the participants to understand the questions. This led to the revision of some of the items in scale A and the inclusion of some additional items. Drawing on the initial item pool and inspired by the HLS framework [15] and MOHLAA-Q [35], we changed some of the items to reflect the following two distinct dimensions of HL: health promotion and disease prevention. For example, the question “…decide whether a source of health information can be trusted” (chl4) was split into the following three more specific questions: “…decide whether media information about healthy habits can be trusted”, “…decide whether media information about diseases can be trusted’”, and “…decide whether information about unhealthy habits can be trusted”. Two of the items (chl1, chl6) in scale A cross-loaded on the two factors. Item chl6 focuses on the ability to recognize when information is also an advertisement. In the revised version, this was changed to address only the dimension of health promotion. This change was made based on the expectation that adolescents in the school context are less likely to be exposed to commercials relating to disease prevention. Another change was made to scale A based on problems with chl1 that were detected in the EFA. The interviewees confirmed that they considered comparing information from different sources (chl1) and seeking help (chl7) to be important strategies. However, these items are arguably different from the other items in the piloted version of scale A because they target specific strategies for information appraisal. The EFA also showed that chl1 had substantial cross-loading and low factor loadings. To accommodate this, the opening phrase for these items was changed from “…how easy or difficult is it for you to…” to “…I am a person who…”. Since the items relate to specific strategies, it might be easier for young people to state whether they normally apply these strategies, rather than to judge how easy it is to implement these strategies. Two more items were added with this new opening phrase to bring balance to the number of items in the factors. The revised version of scale A has two parts, scale A1 and scale A2. These scales address the following three hypothesized factors: the perceived difficulty of judging the accuracy of health information, the perceived difficulty of judging the relevance of health information, and self-efficacy for judging the accuracy of health information and seeking help (see Table 4).

The adolescents understood the items in scale B, but the participants reported that it was difficult to say whether they agreed or disagreed with some of the statements. It was suggested that a fifth middle option should be added to allow more neutral and therefore more honest answers. Thus, the four options, which ranged from completely disagree to completely agree, were expanded to five options with the addition of the middle option, “slightly agree”. The participants reported that the items in scale C were comprehensible and that most were relevant to them. Thus, no changes were made to this scale based on the post-survey interviews. Table 5 presents the key content of the three domains.

## 4. Discussion

Important recommendations for successful interventions to improve HL include the whole school approach, collaboration with school-based health services, inquiry-based critical pedagogy, and teaching HL across different subjects [4]. CHL is one recommended approach for developing HL in school contexts [4,6,20]. The new cross-curricular subject HLS provides an appropriate setting for implementing these recommendations in the Norwegian school. However, few interventions to improve CHL in schools have reported evaluative outcomes [24]. This could be due to a lack of consistent operationalization of the concept; it may also be because no measurement tool that covers the three distinct domains of CHL is available [24]. Adequate measurement is an important part of planning and executing a successful intervention [56], which in turn can promote CHL and ultimately lead to better health and well-being among adolescents.

This study reports the first two stages of developing a self-reported questionnaire (CHLA-Q) that includes three scales to measure adolescents’ critical information appraisal skills (scale A), awareness of the social determinants of health (scale B), and citizenship skills to promote health and well-being in a collective (scale C). The results indicate that these distinct areas of CHL are meaningful topics that adolescents understand and find relevant to their daily lives. This study contributes to identify key aspects within each domain of CHL. The quantitative results of the cross-sectional survey (step 6) were used to delete and adjust items that did not perform satisfactorily. Considering these data, together with the findings of the post-survey focus group interviews, we further revised scales A and B. The third stage in scale development is scale evaluation [36]. Therefore, future research should test the current version of CHLA-Q with a larger sample to allow for more robust psychometric testing.

The versions of scales A and C that were tested in the quantitative phase (step 6) of this study showed good internal reliability, and their fit indices were promising. Convergent validity was examined by testing a pre-determined hypothesis that the sum scores for each scale should positively correlate with generic subjective HL, HrQoL, and self-reported health status. This hypothesis was confirmed for scales A and C and, thus, aligns with previous research. For example, Domanska et al. [35] found that generic HL and subjective health status correlated with their scales on “dealing with health-related information” and “social interaction skills”; these scales are roughly parallel to scale A and scale C in our study, and the degrees of correlation in that study are also similar to ours. Other studies have found a positive association between generic HL and quality of life [48,57].

We found no significant correlations between the pilot version of scale B and HL, HrQoL, or subjective health status. There are several possible explanations for this. There may be no association in our sample or in the target population in general; however, it is also possible that the items piloted in this study did not actually measure the intended construct. Furthermore, the internal reliability of scale B (Cronbach’s α = 0.61) was low, and it is perhaps problematic to use the sum score of this scale, as we did in the correlation tests [45]. However, low reliability is not uncommon in relatable multidimensional HL measurement [29,30,35]. For example, the scale measuring attitudes towards one’s own health and health information in the MOHLAA-Q questionnaire has similarly low reliability in a sample of German adolescents [35]. Initially, scale B included three items measuring awareness of individual versus collective responsibility for health (chl10, chl11, chl15). These items were all excluded based on poor psychometric properties in item analysis and EFA (step 6). The remaining items target awareness of social inequalities in health, which may not encompass the full extent of this theoretical domain [21]. Still, it could be argued that recognizing that people have different health opportunities implies an understanding that health is determined by more than individual choices. In the post-survey focus group interviews, the respondents reported that it was difficult to choose one alternative (agree or disagree), because they did not completely agree or disagree with these statements. Previous research on lay understandings of SDH has shown that, despite a tendency to attribute health outcomes to individual choices, most people favor more complex explanations [58,59,60]. With only four response options, respondents must agree or disagree, and this dichotomizes a concept that most people consider more complex. In the revised version, a fifth response option was added to this scale to allow the respondents to express a more neutral stance [38]. It remains to be seen whether these changes improve the scale, but results from the pilot survey weakens support for the hypothesis that awareness of social determinants of health correlates positively with HL, HrQoL, and/or self-reported health status.

The results of the focus group interviews (step 2) indicate that even young adolescents (13–15 years old) can understand complex relationships between social structures and health outcomes. This is crucial to the theoretical framework of CHL [21,55,61]. However, we found no significant correlation of scale B with scale A or scale C. This result indicates that awareness of SDH is not part of a multidimensional CHL construct at the individual level, but more research is needed to confirm or refute this.

### Limitations and Future Research

This study has several limitations. We only included self-reported indicators, although it is recommended to include both objective and subjective indicators in HL measurement [26]. There are, however, several advantages to self-reported indicators. They are easy to administer and less burdensome to complete [62]. Furthermore, it is easier to include different dimensions in such measurements, while performance-based items are significantly impacted by participants’ functional skills. This was considered very important since HL and CHL are complex, multidimensional social constructs [10,21]. Moreover, self-reported indicators can predict behavior [63], which is an important endpoint for research on CHL. Still, performance-based indicators are associated with less measurement error [56], and a combination of different indicators may well lead to higher overall measurement quality. We chose to prioritize adolescents’ perspectives and minimize the burden of participation in the study by preparing a simple, short, self-reported questionnaire.

One strength of the present study is that the results of our qualitative investigation align with the findings from parallel research on health-related information appraisal [54] and on the social determinants of health domains [58,59,60], even though the latter studies involved adult participants. The convergent validity analysis also show that scales A and C perform as expected and in accordance with similar research in other populations [35,48,57]. However, these findings must be tested again due to the small sample size in the present study and the revisions made to scales A and B after the survey.

During all steps of this study, participant selection was based on convenience and willingness to participate, which could lead to a skewed selection of the population. Therefore, the present findings cannot be generalized beyond the sample in this study. Further testing of the revised set of items is necessary to determine the psychometric properties and validity of the measurement tool. In the next step, the questionnaire will be distributed to a larger sample; this will allow more robust psychometric testing, including confirmatory factor analysis and item response theory [36]. It is also necessary to investigate how the items function across different demographic groups (e.g., age and gender). Future research on CHL in lower secondary schools should investigate the relationships of the domains of CHL with generic health literacy, quality of life, subjective health, and other factors that are related to health and well-being in the school context.

CHL is a complex concept that is difficult to operationalize; this is particularly true for the domains represented in scale B and C of this study. In previous research on adolescents, CHL has usually been addressed as one level of HL and assessed in terms of critical information appraisal [29,30,31,32,33,34,35]. We aimed to operationalize and develop indicators for all three domains of CHL, but due to the complexity and scope of this study, we cannot be sure that all aspects of the domains are addressed. We had to select certain attributes as the main components of our scales, and we recognize that despite the thorough development process described in this paper, there may be aspects of CHL that we do not cover in the revised version of CHLA-Q.

## 5. Conclusions

The cross-curricular subject Health and Life Skills provides a convenient setting for developing adolescents’ HL and CHL in Norway. Health education in schools that increase adolescents CHL is of great importance in modern society and particularly considering the recent COVID-19 pandemic. Adequate measurement is necessary to provide knowledge that can guide and evaluate such efforts. To our knowledge, this is the first study to measure self-reported CHL in the school context. The scales B and C in our study contribute to the knowledge on adolescent’s awareness of social determinants of health and citizenship for health and well-being, aspects that are particularly important for HL in relation to social responsibility and solidarity. These domains are not addressed explicitly in previous measurement of HL or CHL for adolescents, and thus our study provides a notable theoretical contribution. However, there are several limitations with our study, and more in-depth research is needed before the questionnaire can be applied. The insights gained from this initial development and testing of the CHLA-Q should be utilized to further develop and strengthen the psychometric properties of this questionnaire so that it can be used to inform, tailor, and evaluate future interventions to enhance adolescents’ CHL, health, and well-being.

## Figures and Tables

**Table 1:** Exploratory factor analysis for scale A. Factor loadings < 0.30 are suppressed. h2 = communality (amount of variance explained by common factors). u2 = residual variance (variance unique to the variable). com = indicator of cross-loading.

Scale A: Critical Information Appraisal					
Items	F1	F2	h2	u2	com
chl9	0.86		0.74	0.26	1.0
chl8	0.76		0.66	0.34	1.0
chl7	0.68		0.38	0.62	1.1
chl5		0.90	0.77	0.23	1.0
chl4		0.68	0.54	0.46	1.0
chl6		0.40	0.31	0.69	1.6
chl1		0.33	0.27	0.73	1.9
Eigenvalue	2.0	1.7			
Prop var	0.28	0.24			
Cronbach’s α = 0.83 (α_F1_ = 0.80, α_F2_ = 0.75)					
TLI = 0.97 RSMEA = 0.055 [CI 90%, 0–0.132]					

F1: perceived ease of judging relevance and seeking help. F2: perceived ease of judging accuracy.

**Table 2:** Exploratory factor analysis of scale B. Factor loadings < 0.30 are suppressed. h2 = communality (amount of variance explained by common factors). u2 = residual variance (variance unique to the variable). com = indicator of cross-loading.

Scale B: Awareness of Social Determinants of Health				
Items	F1	h2	u2	com
chl12	0.80	0.63	0.37	1
chl14	0.37	0.13	0.87	1
chl16	0.55	0.30	0.70	1
chl13	0.46	0.21	0.79	1
Eigenvalue	1.3			
Prop var	0.32			
Cronbach’s α = 0.61				
TLI = 1.05 RMSEA = 0.00 [CI 90%, 0–0.16]				

**Table 3:** Exploratory factor analysis for scale C. Factor loadings < 0.30 are suppressed. h2 = communality (amount of variance explained by common factors). u2 = residual variance (variance unique to the variable). com = indicator of cross-loading.

Scale C: Citizenship for Health and Well-Being					
Items	F1	F2	h2	u2	com
chl26	0.98		0.87	0.13	1.0
chl24	0.70		0.69	0.31	1.2
chl20	0.63		0.54	0.46	1.2
chl25	0.52		0.46	0.54	1.4
chl28		0.71	0.44	0.56	1.1
chl22		0.57	0.39	0.61	1.1
chl18		0.56	0.45	0.55	1.2
chl23		0.56	0.43	0.57	1.2
Eigenvalue	2.4	1.8			
Prop var	0.30	0.23			
Cronbach’s α = 0.85 (α_F1_ = 0.86, α_F2_ = 0.73) TLI = 0.95 RMSEA = 0.074 [CI 90%, 0–0.13]					

F1: self-efficacy in providing social support. F2: self-efficacy in democratic participation to promote health and well-being.

**Table 4:** Revised items and scales of the CHLA-Q.

Revised or Added in Step 7	Source	Scale A1: Critical Information Appraisal How Easy or Difficult Is It for You to…	Component
(chl4) revised	MOHLAA-Q [35]	…decide whether media information about illness can be trusted (media: internet, TV, newspapers)	Accuracy/impartiality
(chl6) revised	Self-developed	…decide whether information about healthy habits is also a commercial (healthy habits: what you eat/drink, physical activity)	Accuracy/impartiality
(chl4) revised	MOHLAA-Q	…decide whether media information about healthy habits can be trusted (healthy habits: what you eat/drink, physical activity)	Accuracy/impartiality
(chl8) revised	Self-developed	…find out whether health information is relevant to you when you are ill	Relevance
New	HLS-Child-Q15 [34]	…judge what food is healthy for you	Relevance
(chl9) revised	Self-developed	…judge whether information on healthy habits is relevant to you (healthy habits: what you eat/drink, physical activity)	Relevance
(chl4) revised	MOHLAA-Q	…judge whether information about unhealthy habits can be trusted (unhealthy habits: alcohol, tobacco, what you eat/drink)	Accuracy/impartiality
Revised or added in step 7	Source	Scale A2: Critical information appraisal I am a person who…	
(chl1) revised	HELMA [30]	…compares health information from different sources (sources: internet, parents, friends)	Accuracy/impartiality
New	HLS-Child-Q15	…can decide what I need to do to stay healthy and what doesn’t help that much	Accuracy/impartiality
(chl5) revised	HELMA	…can decide whether a source of health information can be trusted (source: internet, TV, newspapers)	Accuracy/impartiality
(chl7) revised	Self-developed	…asks for help if I am unsure about the trustworthiness of health information	Accuracy/impartiality/Help seeking
Revised or added in step 7	Source	Scale B: Awareness of social determinants of health To what extent do you agree with the following statements?	
(chl12) unchanged	Self-developed	Some groups in society have fewer opportunities for good health.	Health inequality
(chl13) unchanged	Self-developed	Everybody has the same opportunities to be healthy.	Health inequality
(chl14) revised	Self-developed	Where you grow up could have a significant impact on your health.	Health inequality
(chl16) unchanged	Self-developed	It is unfair that some groups in society have poorer health than others.	Health inequality
Revised or added in step 7 (revised/new)		Scale C: Citizenship for health and well-being I am a person who…	
(chl26) unchanged	UiL/self-developed [53]	…can support others if they are feeling sad.	Citizenship/social support
(chl24) unchanged	UiL/self-developed	…can help others if they are not doing well.	Citizenship/social support
(chl20) unchanged	Self-developed	…can contribute to the well-being of others in my class.	Citizenship/social support
(chl25) unchanged	UiL	…can help find solutions that are acceptable to all parties.	Citizenship/social support
(chl28) unchanged	Self-developed	…can easily talk to others, even if I don’t know them very well.	Citizenship/democratic participation
(chl18) unchanged	HELMA	…can share information about factors that influence health with others.	Citizenship/democratic participation
(chl23) unchanged	Self-developed	…believes my knowledge about health could be useful for others.	Citizenship/democratic participation
(chl22) unchanged	HLSAC [32]	…is aware of how my actions can influence others (e.g., attitude, mood).	Citizenship/democratic participation

**Table 5:** Key content of measurement domains.

Measurement Domain	Key Content	
Scale A Critical information appraisal of health-related information	Appraisal leads to decisions about the credibility and relevance of health-related information. Important criteria adolescents can use include the extent to which a piece of information is accurate, impartial, and relevant to them [33]. During appraisal, adolescents investigate the characteristics of information (e.g., who wrote it, when it was published, and what sources the writer used) and compare information from different sources. They apply prior knowledge and seek help [54]. Sometimes they feel helpless.	(A1) Perceived difficulty of performing cognitive tasks. (A2) Self-efficacy to perform cognitive tasks
Scale B Awareness of social determinants of health (SDH)	Awareness of SDH involves a deeper understanding of health and encourages an understanding that health is influenced by more than individual lifestyle choices [21]. Recognizing health inequities is crucial to understanding the causes behind these inequities [55]	Attitudes/self-reported knowledge
Scale C Citizenship for health and well-being in the school context	Citizenship in HL centers on the ability to understand and act on the rights and responsibilities that come with participation in a democratic collective [1]. Key components are self-efficacy to provide social support and participate in democratic discussions on matters that concern health and well-being.	Self-efficacy to perform social and communicative tasks

## Data Availability

Not applicable.

## Supplementary Materials

The following supporting information can be downloaded from: https://www.mdpi.com/article/10.3390/ijerph19053116/s1. Table S1: Descriptive statistics for each item of the piloted version of the CHLA-Q (second draft). Table S2: List of studies and items from LR [29,30,31,32,33,34,35].

## References

[1] Paakkari L., Paakkari O. (2012). Health literacy as a learning outcome in schools. Health Educ..

[2] WHO (2013). Health 2020 A European Policy Framework and Strategy for the 21st Century. https://www.euro.who.int/__data/assets/pdf_file/0011/199532/Health2020-Long.pdf.

[3] WHO (2016). Shanghai Declaration on Promoting Health in the 2030 Agenda for Sustainable Development. https://www.who.int/healthpromotion/conferences/9gchp/shanghai-declaration.pdf.

[4] Nash R., Patterson K., Flittner A., Elmer S., Osborne R. (2021). School-Based Health Literacy Programs for Children (2–16 Years): An International Review. J. Sch. Health.

[5] Paakkari L., Okan O. (2020). COVID-19: Health literacy is an underestimated problem. Lancet Public Health.

[6] Peralta L., Rowling L., Samdal O., Hipkins R., Dudley D. (2017). Conceptualising a new approach to adolescent health literacy. Health Educ. J..

[7] WHO Health Literacy in the Context of Health, Well-Being and Learning Outcomes-the Case of Children and Adolescents in Schools. https://www.euro.who.int/en/health-topics/Life-stages/child-and-adolescent-health/publications/2021/health-literacy-in-the-context-of-health,-well-being-and-learning-outcomes-the-case-of-children-and-adolescents-in-schools-the-case-of-children-and-adolescents-in-schools-2021.

[8] Lovdata https://lovdata.no/dokument/NLE/lov/1998-07-17-61.

[9] Norwegian Directorate for Education and Training. https://www.udir.no/lk20/overordnet-del/?lang=eng.

[10] Schulenkorf T., Sørensen K., Okan O. (2022). International Understandings of Health Literacy in Childhood and Adolescence— A Qualitative-Explorative Analysis of Global Expert Interviews. Int. J. Environ. Res. Public Health.

[11] Nutbeam D. (2000). Health literacy as a public health goal: A challenge for contemporary health education and communication strategies into the 21st century. Health Promot. Int..

[12] Nutbeam D. (2008). The evolving concept of health literacy. Soc. Sci. Med..

[13] Sørensen K., Orkan O., Bauer U., Levin-Zamir D., Pinheiro P., Sørensen K. (2019). Defining health literacy: Exploring differences and commonalities. International Handbook of Health Literacy. Research, Practice and Policy across the Lifespan.

[14] Nutbeam D. (1998). Health Promotion Glossary. Health Promot. Int..

[15] Sorensen K., van den Broucke S., Pelikan J.M., Fullam J., Doyle G., Slonska Z., Kondilis B., Stoffels V., Osborne R.H., Brand H. (2013). Measuring health literacy in populations: Illuminating the design and development process of the European Health Literacy Survey Questionnaire (HLS-EU-Q). BMC Public Health.

[16] Broder J., Okan O., Bauer U., Bruland D., Schlupp S., Bollweg T.M., Saboga-Nunes L., Bond E., Sorensen K., Bitzer E.M. (2017). Health literacy in childhood and youth: A systematic review of definitions and models. BMC Public Health.

[17] Bröder J., Okan O., Bauer U., Schlupp S., Pinheiro P. (2020). Advancing perspectives on health literacy in childhood and youth. Health Promot. Int..

[18] Paakkari L., George S. (2018). Ethical underpinnings for the development of health literacy in schools: Ethical premises (‘why’), orientations (‘what’) and tone (‘how’). BMC Public Health.

[19] Madsen O.J. (2020). Livsmestring på Timeplanen: Rett Medisin for Elevene? [Life Skills on the Curricula–Prescription for Healthy Pupils?].

[20] Ryan M., Rossi T., Hunter L., Macdonald D., McCuaig L. (2012). Theorising a Framework for Contemporary Health Literacies in Schools. Curric. Perspect..

[21] Chinn D. (2011). Critical health literacy: A review and critical analysis. Soc. Sci. Med..

[22] Sykes S., Wills J., Rowlands G., Popple K. (2013). Understanding critical health literacy: A concept analysis. BMC Public Health.

[23] Sykes S., Wills J. (2018). Challenges and opportunities in building critical health literacy. Glob. Health Promot..

[24] Sykes S., Wills J. (2019). Critical health literacy for the marginalised: Empirical findings. International Handbook of Health Literacy-Research, Practice and Policy across the Lifespan.

[25] Guo S.J., Armstrong R., Waters E., Sathish T., Alif S.M., Browne G.R., Yu X.M. (2018). Quality of health literacy instruments used in children and adolescents: A systematic review. BMJ Open.

[26] Okan O., Lopes E., Bollweg T.M., Bröder J., Messer M., Bruland D., Bond E., Carvalho G.S., Sørensen K., Saboga-Nunes L. (2018). Generic health literacy measurement instruments for children and adolescents: A systematic review of the literature. BMC Public Health.

[27] Ormshaw M.J., Paakkari L.T., Kannas L.K. (2013). Measuring child and adolescent health literacy: A systematic review of literature. Health Educ..

[28] Perry E.L. (2014). Health literacy in adolescents: An integrative review. J. Spec. Pediatric Nurs..

[29] Abel T., Hofmann K., Ackermann S., Bucher S., Sakarya S. (2015). Health literacy among young adults: A short survey tool for public health and health promotion research. Health Promot. Int..

[30] Ghanbari S., Ramezankhani A., Montazeri A., Mehrabi Y. (2016). Health Literacy Measure for Adolescents (HELMA): Development and Psychometric Properties. PLoS ONE.

[31] Manganello J.A., DeVellis R.F., Davis T.C., Schottler-Thal C. (2015). Development of the Health Literacy Assessment Scale for Adolescents (HAS-A). J. Commun. Healthc..

[32] Paakkari O., Torppa M., Kannas L., Paakkari L. (2016). Subjective health literacy: Development of a brief instrument for school-aged children. Scand. J. Public Health.

[33] Wu A.D., Begoray D.L., MacDonald M., Higgins J.W., Frankish J., Kwan B., Fung W., Rootman I. (2010). Developing and evaluating a relevant and feasible instrument for measuring health literacy of Canadian high school students. Health Promot. Int..

[34] Bollweg T.M., Okan O., Pinheiro P., Bauer U. (2016). Development of a health literacy measurement tool for primary school children in Germany. Eur. J. Public Health.

[35] Domanska O.M., Bollweg T.M., Loer A.K., Holmberg C., Schenk L., Jordan S. (2020). Development and Psychometric Properties of a Questionnaire Assessing Self-Reported Generic Health Literacy in Adolescence. Int. J. Environ. Res. Public Health.

[36] Boateng G.O., Neilands T.B., Frongillo E.A., Melgar-Quiñonez H.R., Young S.L. (2018). Best Practices for Developing and Validating Scales for Health, Social, and Behavioral Research: A Primer. Front. Public Health.

[37] Robson C. (2002). Real World Research: A Resource for Social Scientists and Practitioner-Researchers.

[38] DeVellis R.F. (2003). Scale Development-Theory and Applications.

[39] Walseth K. (2016). Literacies for Health and Life Skills. https://uni.oslomet.no/hls/.

[40] Friborg O., Martinussen M. (2010). Klassisk testteori og utvikling av spørreinstrumenter [Classical test theory and development of questionaires]. Kvantitativ Forskningsmetodologi i Samfunns-og Helsefag [Quantitative Research Methodology in Social-and Health Sciences].

[41] Ulleberg P., Nordvik H. (2001). Faktoranalyse [Factor Analysis].

[42] Dziuban C.D., Shirkey E.C. (1974). When is a correlation matrix appropriate for factor analysis? Some decision rules. Psychol. Bull..

[43] Kaiser H.F., Rice J. (1974). Little Jiffy, Mark Iv. Educ. Psychol. Meas..

[44] Browne M.W., Cudeck R. (1992). Alternative Ways of Assessing Model Fit. Sociol. Methods Res..

[45] Terwee C.B., Bot S.D.M., de Boer M.R., van der Windt D.A.W.M., Knol D.L., Dekker J., Bouter L.M., de Vet H.C.W. (2007). Quality criteria were proposed for measurement properties of health status questionnaires. J. Clin. Epidemiol..

[46] Paakkari O., Torppa M., Boberova Z., Valimaa R., Maier G., Mazur J., Kannas L., Paakkari L. (2019). The cross-national measurement invariance of the health literacy for school-aged children (HLSAC) instrument. Eur. J. Public Health.

[47] Bjørnsen H.N., Ringdal R., Espnes G.A., Eilertsen M.-E.B., Moksnes U.K. (2018). Exploring MEST: A new universal teaching strategy for school health services to promote positive mental health literacy and mental wellbeing among Norwegian adolescents. BMC Health Serv. Res..

[48] Riiser K., Helseth S., Haraldstad K., Torbjørnsen A., Richardsen K.R. (2020). Adolescents’ health literacy, health protective measures, and health-related quality of life during the COVID-19 pandemic. PLoS ONE.

[49] Haraldstad K., Christophersen K.-A., Eide H., Nativg G.K., Helseth S. (2011). Health related quality of life in children and adolescents: Reliability and validity of the Norwegian version of KIDSCREEN-52 questionnaire, a cross sectional study. Int. J. Nurs. Stud..

[50] Fayers P.M., Sprangers M.A.G. (2002). Understanding self-rated health. Lancet.

[51] Joffer J., Jerden L., Ohman A., Flacking R. (2016). Exploring self-rated health among adolescents: A think-aloud study. BMC Public Health.

[52] Revelle W. (2021). Psych: Procedures for Psychological, Psychometric, and Personality Research [R Package Version 2.1.9].

[53] Makransky G., Wandall J., Madsen S.R., Hood M., Creed P. (2020). Development and Validation of the UiL-Scales for Measurement of Development in Life Skills—A Test Battery of Non-Cognitive Skills for Danish School Children. Scand. J. Educ. Res..

[54] Stars I., Rubene Z. (2020). A Phenomenographic Study of Adolescents’ Conceptions of Health Information Appraisal as a Critical Component of Adolescent Health Literacy. Acta Paedagog. Vilnensia.

[55] Mogford E., Gould L., DeVoght A. (2010). Teaching critical health literacy in the US as a means to action on the social determinants of health. Health Promot. Int..

[56] Pleasant A. (2014). Advancing Health Literacy Measurement: A Pathway to Better Health and Health System Performance. J. Health Commun..

[57] Ran M., Peng L., Liu Q., Pender M., He F., Wang H. (2018). The association between quality of life(QOL) and health literacy among junior middle school students: A cross-sectional study. BMC Public Health.

[58] Popay J., Bennett S., Thomas C., Williams G., Gatrell A., Bostock L. (2003). Beyond ‘beer, fags, egg and chips’? Exploring lay understandings of social inequalities in health. Sociol. Health Illn..

[59] Blaxter M. (1997). Whose fault is it? People’s own conceptions of the reasons for health inequalities. Soc. Sci. Med..

[60] Davidson R., Kitzinger J., Hunt K. (2006). The wealthy get healthy, the poor get poorly? Lay perceptions of health inequalities. Soc. Sci. Med..

[61] Gould L., Mogford E., DeVoght A. (2010). Successes and Challenges of Teaching the Social Determinants of Health in Secondary Schools: Case Examples in Seattle, Washington. Health Promot. Pract..

[62] Beitchman J.H., Corradini A. (1988). Self-report measures for use with children: A review and comment. J. Clin. Psychol..

[63] Bandura A. (2004). Health Promotion by Social Cognitive Means. Health Educ. Behav..

