# Supplementary material for: Developing Indicators to Measure Critical Health Literacy in the Context of Norwegian Lower Secondary Schools
Source: Int J Environ Res Public Health. 2022 Mar 7;19(5):3116. doi: 10.3390/ijerph19053116 (PMC8910382; doi:10.3390/ijerph19053116)
Supplement: Supplementary file 1 [file ijerph-19-03116-s001.zip › ijerph-1608544-supplementary.pdf]

Table S1: Descriptive statistics for each item on the piloted version of the CHLA-Q (second draft)

Var name	Scale A Information appraisal<br>How easy or difficult is it for you to…	N	Mean	SD	Kurtosis	Variance	Skew	Chronbach's α if item is dropped	ITC
**chl1**	…compare information about health from different sources	113	3.21	0.91	-0.17	0.76	-0.21	0.85	0.52
**chl2**	…find out if health related information is right of wrong	114	3.03	0.88	0.22	0.74	0.18	0.85	0.63
**chl3**	…decide if health related information is true	111	2.96	0.74	-0.11	0.52	-0.08	0.85	0.56
**chl4**	…find out if health related information can be trusted	114	3.30	0.90	-0.09	0.78	-0.47	0.85	0.60
**chl5**	…decide if a source of health information can be trusted	113	3.27	0.78	0.31	0.63	-0.18	0.85	0.61
**chl6**	…recognize information about health that is also a commercial	114	3.37	0.96	-0.32	0.94	-0.49	0.86	0.50
**chl7**	…ask for help if you doubt that health information can be trusted	114	3.68	0.99	0.45	0.92	-0.7	0.86	0.43
**chl8**	…find out if health related information fits you in the current situation	113	3.31	0.92	-0.32	0.86	-0.3	0.85	0.66
**chl9**	…judge if health information applies to your context	114	3.26	0.88	0.03	0.80	-0.38	0.85	0.65
	**Scale B Awareness of social determinants of health.**<br>To what degree to you agree with the following statements?	**N**	**Mean**	**SD**	**Kurtosis**	**Variance**	**Skew**	**Chronbach's α if item is dropped**	**ITC**
**chl10**	I am mostly responsible for my own health	113	3.22	0.55	1.44	0.30	-0.24	0.57	0.05
**chl11**	A healthy lifestyle is the most important thing for å persons health	114	3.00	0.65	-0.67	0.43	0.00	0.53	0.19
**chl12**	Some groups in society have poorer opportunities for good health	112	3.12	0.65	-0.71	0.44	-0.12	0.43	0.51
**chl13**	Everybody has the same opportunities for good health	113	2.80	0.85	-0.16	0.68	-0.57	0.53	0.22
**chl14**	Where you grow up could have a significant impact on your health	112	3.11	0.63	0.22	0.37	-0.30	0.47	0.37
**chl15**	Health is determined by where you live, but people can choose to live wherever they want	111	2.68	0.70	-0.26	0.49	-0.09	0.56	0.09
**chl16**	It is unfair that some groups in society have poorer health than others	113	3.01	0.77	-0.44	0.59	-0.36	0.48	0.35
**chl17**	Good opportunities for education and work are important for health	114	3.04	0.55	0.20	0.28	0.02	0.50	0.35
	**Citizenship for health and well-being.**<br>**I am a person that…**	**N**	**Mean**	**SD**	**Kurtosis**	**Variance**	**Skew**	**Chronbach's α if item is dropped**	**ITC**
**chl18**	…can share information on factors that influence health with others	114	3.63	0.91	-0.04	0.88	-0.46	0.87	0.62
**chl19**	…can discuss my worries about health with others	113	3.39	0.99	-0.36	1.01	-0.39	0.87	0.52
**chl20**	…can contribute to the well-being of others in my class	113	3.92	0.79	2.16	0.65	-1.04	0.86	0.63
**chl21**	…can make sure that my ideas get executed (e.g., in cooperation with others)	114	3.43	0.91	0.25	0.84	-0.38	0.87	0.62
**chl22**	…am aware of how my actions can influence others (e.g., attitude, mood)	112	3.98	0.77	1.03	0.60	-0.67	0.87	0.54
**chl23**	…believe my knowledge on health could be useful for others	113	2.88	0.86	-0.08	0.73	-0.19	0.87	0.61
**chl24**	…can help others if they are feeling bad	112	4.09	0.79	1.44	0.61	-0.92	0.86	0.71
**chl25**	…can help to find solutions that is acceptable to all parties	114	3.55	0.8	0.6	0.67	-0.27	0.86	0.64
**ch**l26	…can support others if they are feeling sad	114	4.28	0.81	3.39	0.68	-1.52	0.87	0.63
**chl27**	…listen to what others know about health (e.g., friends, classmates)	113	3.90	0.78	1.32	0.61	-0.84	0.87	0.57
**chl28**	…easily can talk to others, even if I don't know them to well	114	3.01	1.04	-0.58	1.09	0.12	0.88	0.44

Table S2. List studies and items from LR.

Instrument	Short name	Items targeting domains of critical health literacy	Factor/component as descriped in original article	Relevant to specific domain of CHL	Short description of instrument:	References
**The Health Litearcy Assesment Scale**	HAS-A	How often do you get confused because you find different information about the same health topic?	Process / Confusion	Information appraisal	The Health Literacy Assessment Scale for adolescents (HAS-A) is based on a framework of four areas within health literacy, that is obtain, understand, communicate and process health information (Manganello, DeVellis, Davis, & Schottler-Thal, 2015). After a factor analysis to identify self-report items three factors emerged, that is functional health literacy, communication, and confusion. The measure focuses on the health care setting, but also include items more related to health promotion. The confusion domain separates this measure a little from other tools described here. Some items here might be negatively correlated with the appraisal or action domain in the CHL framework, that is if a person gets confused from different information, that is an indication of low levels of critical appraisal skills. The wording of the item is, how often do you get confused because you find different information about the same health topic? Furthermore, the study provides some evidence that self-reported HL is correlated with performance-based HL because all self-reported items are compared with a "gold-standard" performance-based items from REALM-teen and NVS (Manganello et al., 2015)	[1]
**Measurment of Health Literacy Among Adolescents Questionnaire**	MOHLAA-Q	How easy or difficult is it for you to… judge which information about an illness in the media you can trust and which you cannot	Dealing with health-related information	Information appraisal	The Measurement of Health Literacy Among Adolescents Questionnaire (MOHLAA-Q) are recently developed as an age adjusted generic instrument for adolescents aged 14-17 years in Germany (Domanska et al., 2020). In the process the HLS-EU-Q47-GER was used as a blueprint and, based on qualitative and quantitative investigations items where modified and adapted to the target group. Final tool consisted for four scales with 29	[2]

		(media: internet, TV, radio, press)			items in three areas: (A) dealing with health-related information, (B) interaction and communication skills, (C) Attitudes towards one's own health and health information. Critical appraisal is measured through three items in scale A, asking participants to respond to how difficult it is for you to: (info 4) judge which information about an illness in the media you can trust and which you cannot (media: internet, TV, radio, press), (info 8) judge whether you can trust the media when they warn you of health risks, (info 13) judge how what you do daily affects your health (e. g. eating, drinking, exercise, relaxation, body care).	
	MOHLAA-Q	judge whether you can trust the media when they warn you of health risks	Dealing with health-related information	Information appraisal		
	MOHLAA-Q	judge how what you do daily affects your health (e. g. eating, drinking, exercise, relaxation, body care).	Dealing with health-related information	Information appraisal		
	HLAT-8	How well are you doing in choosing the advices and offers that fit with you the most?	Critical level of health literacy	Information appraisal	Abel, Hofmann, Ackermann, Bucher, and Sakarya (2015) developed a short instrument of health literacy for family and private contexts based on population survey data in Switzerland (HLAT-8). The measure includes 8 items and three dimensions, four measuring functional HL, and two items each for interactive and critical HL. A strength with HLAT-8 is that it is short, and easy to distribute, it also prompts the respondents to relate items to a family and friends realm, which is essential for HL in childhood and adolescence (Guo et al., 2018). The two items measuring critical HL are: (HL7) How well are you doing in choosing the advice and offers that fit with you the most? and (HL8) regarding information on health on the Internet, I'm able to determine which of the sources are of high and which of poor quality.	[3]
	HLAT-8	regarding information on health on the Internet, I'm able to determine which of the sources are of high and which of poor quality	Critical level of health literacy	Information appraisal		
**The Health Literacy Measure for Adolescents**		*When faced with new health information...*			The Health Literacy Measure for Adolescents (HELMA) was developed for adolescents aged 15-18 years old in Iran (Ghanbari, Ramezankhani, Montazeri, & Mehrabi, 2016). It is a multidimensional comprehensive measure with eight factors and 44 items, items are not evenly distributed between factors, ranging from 10 items in understanding to 3 items in numeracy. The numeracy items are performance based, while the other factors are self-reported. HELMA showed good overall internal consistency (Cronbachs α = 0.93). However, some of the subscales did not. The critical aspect is measured through appraisal in five items; When faced with new health information (25) I can jugde its accuracy, (26) I would compare the data obtained from various sources, (27) When dealing with conflicting information about health issues I can recognize the correct information, (28)	[4]
	HELMA	I can jugde its accuracy	Appraisal	Information appraisal		
	HELMA	I would compare the data obtained from various sources	Appraisal	Information appraisal		
	HELMA	When dealing with conflicting information about health issues I can recognize the correct information	Appraisal	Information appraisal		

	HELMA	I have the ability to jugde which resources I can trust	Appraisal	Information appraisal	I have the ability to jugde which resources I can trust, and (29) when dealing with nutritional information I can choose the right information.	
	HELMA	When dealing with nutritional information I can choose the right information	Appraisal	Information appraisal		
	HELMA	I can share the health information that I gather with others (e.g. family, friends, etc.)	Communication	Collective action for health and well-being		
**European Health Literacy Survey Questionnaire for Children**		*How easy or difficult is it for you to…*			HLS-Child-Q15 was adapted for children aged 9-10 years (T. M. Bollweg, Okan, Pinheiro, & Bauer, 2016). In total 26 items were modified and distributed to a sample of German children. Eleven items were deleted in the psychometric analysis and the final instrument consisted of 15 items measuring generic HL, of which one item was intended to measure the appraisal dimension.	[5]
	HLS-Child-Q15	judge what helps a lot for you to stay healthy and what does not help much?	Appraisal/understanding	Information appraisal		
	HLS-Child-Q15	find out which food is healthy for you?	Access	Information appraisal		
**Health Literacy for School-Aged Children**		*I am confident that…*			Another measure that was created for the school context is the Health literacy for school-aged children (HLSAC) (O. Paakkari, Torppa, Kannas, & Paakkari, 2016). It is based on the theoretical framework of HL as learning outcomes for the Finish school context where HL is defined as: *"Health literacy comprises a broad range of competencies that people seek to encompass, evaluate, construct and use. Through health literacy competencies people become able to understand themselves, others and the world in a way that will enable them to make sound health decisions, and to work on and change the factors that constitute their own and others' health chances"* (L. Paakkari & Paakkari, 2012). HLSAC measures subjective HL with ten items in one factor with five theoretically distinct components. These are ordered from the least complex to more advanced: (1) theoretical knowledge, (2) practical knowledge, (3) individual critical thinking, (4) self-awareness and (5) citizenship. Theoretically the components of self-awareness and citizenship are related to the domain for collective action for health, that is the third domain for CHL.	[6]
	HLSAC	I can compare health-related information from different sources	Individual critical thinking	Information appraisal		
	HLSAC	I can jugde how my own actions affect the surrounding natural environment	Citizenship	Collective action for health and well-being		
	HLSAC	When necessary I am able to give ideas on how to improve health in my immediate surroundings (e.g. a nearby place or area, family, friends)	Citizenship	Collective action for health and well-being		

	HLSAC	I can jugde how my behaviour affects my health	Self-awareness	Collective action for health and well-being	Within this framework citizenship evolves around abilities to understand and act upon both the rights and responsibilities that comes with participation in a democratic collective.	
	HLSAC	I can usually figure out if some healht-related information is right or wrong	Individual critical thinking	Information appraisal		
**Health literacy in the Canadian high school context**					Wu et al. (2010) developed a performance-based instrument for measuring HL in Canadian high school students. The instrument consisted of 11 passages of health information followed by two general types of items, that is 30 understand items and 17 evaluate items. The understand items assessed prose, numeracy and document understanding. The evaluate items were distributed across five criterions that can be used to judge the reliability of health information: (1) accuracy, (2) impartiality, (3) relevance, (4) comprehensiveness and (5) internal consistency. A relatively small sample of 275 high school students participated in the study, and principal component analysis was used to confirm the one-dimensionality of the instruments two scales (understand and evaluate). Self-reported health literacy was slightly positively correlated with performance-based health literacy, however it is not clear exactly how this was measured, as it is only listed as self-ratings of HL. Overall, the instrument provides a detailed measurement of student's ability to understand and evaluate health related information, however only including performance-based items related to understanding and evaluating heath information restricts the measure to cognitive and functional abilities. Also, it has been criticized for being time-consuming and difficult to complete (Ghanbari et al., 2016), which is of great importance in a school context.	[7]

A short description of the strategy used in the LR

We found four comprehensive literature reviews and used these as a vantage point (Guo et al., 2018; Okan et al., 2018; Ormshaw, Paakkari, & Kannas, 2013; Perry, 2014). Studies in these reviews were examined and included by the following criterions. (1) Instruments had to be developed for children and adolescents (aged 10-17 years), (2) instruments had to be developed for the general population and for generic HL (domain specific instruments were excluded, e.g. HL in cancer treatment or media health literacy), (3) articles had to be available in English.

In addition, we performed a searches in the following data bases: CINAHL, ERIC, MEDLINE, SPORTDiscus, Education Source, Academic Search Ultimate (through EBSCO-host) and the Web of Science with the following combination of search terms ("Health literacy" OR "critical health literacy" [Ti]) AND (Youth* OR Adolecent* OR Children*) AND (measur* OR test* OR tool* OR instrument* OR questionnaire* OR assessment* OR screen* OR survey* OR psychometric* OR review*) limited to publications between

2018-2020 to identify instruments published after the latest systematic review. Abstracts were scanned and relevant articles included following the stipulated criterions. Two additional instruments were added (Torsten Michael Bollweg et al., 2020; Domanska et al., 2020). The articles identified in this review were read in detail and used for inspiration to adapt and write new items throughout the development process. No further analysis of the articles was done for the purpose of this study.

References

1. Manganello, J. A., DeVellis, R. F., Davis, T. C., & Schottler-Thal, C. (2015). Development of the Health Literacy Assessment Scale for Adolescents (HAS-A). *Journal of Communication in Healthcare*,2015, 8, 172-184. doi:10.1179/1753807615Y.0000000016.
2. Domanska, O. M., Bollweg, T. M., Loer, A. K., Holmberg, C., Schenk, L., & Jordan, S. (2020). Development and Psychometric Properties of a Questionnaire Assessing Self-Reported Generic Health Literacy in Adolescence. *Int J Environ Res Public Health. 2020, 17, 2860. doi: 10.3390/ijerph17082860.*
3. Abel, T., Hofmann, K., Ackermann, S., Bucher, S., & Sakarya, S. (2015). Health literacy among young adults: a short survey tool for public health and health promotion research. Health Promot Int, 30(3), 725-735. doi:10.1093/heapro/dat096
4. Ghanbari, S., Ramezankhani, A., Montazeri, A., & Mehrabi, Y. (2016). Health Literacy Measure for Adolescents (HELMA): Development and Psychometric Properties. PLoS ONE, 11(2), 1-12. doi:10.1371/journal.pone.0149202
5. Bollweg, T. M., Okan, O., Pinheiro, P., & Bauer, U. (2016). Development of a health literacy measurement tool for primary school children in Germany. *Health Lit Res Pract*. 2020, 4, e144–e159.
6. Paakkari, O., Torppa, M., Kannas, L., & Paakkari, L. (2016). Subjective health literacy: Development of a brief instrument for school-aged children. Scandinavian Journal of Public Health, 44(8), 751-757. doi:10.1177/1403494816669639
7. Wu, A. D.; Begoray, D. L.; MacDonald, M.; Wharf Higgins, J.; Frankish, J.; Kwan, B.; Rootman, I. (2010). Developing and evaluating a relevant and feasible instrument for measuring health literacy of Canadian high school students. *Health Promotion International*, 25(4), 444-452. Retrieved from https://login.ezproxy.hioa.no/login?url=http://search.ebscohost.com/login.aspx?direct=true&db=asn&AN=55276133&site=ehost-live
